# Understanding Treatment Adherence in Chronic Diseases: Challenges, Consequences, and Strategies for Improvement

**DOI:** 10.3390/jcm14176034

**Published:** 2025-08-26

**Authors:** Sheena Patel, Mingyi Huang, Sophia Miliara

**Affiliations:** 1 Swedish Orphan Biovitrum AB, 113 64 Stockholm, Sweden; 2 Apellis Pharmaceuticals Inc., Waltham, MA 02451, USA; 3 Sophia Miliara, Norra Stationsgatan 93A, 113 64 Stockholm, Sweden

**Keywords:** chronic disease, medication adherence, medication non-adherence, oral medication, healthcare system barriers, patient education, patient outcomes

## Abstract

Adherence to medications is a significant challenge in chronic disease management. Poor adherence can lead to adverse patient outcomes including disease progression, increased morbidity, reduced quality of life, higher hospitalization rates, increased medical costs, and mortality. Medical adherence is a complex issue, influenced by multiple factors, including patient-related, medication-related, and healthcare system-related barriers. This review explores reasons for both intentional non-adherence, such as patients underestimating the consequences of the disease, inadequate education or poor healthcare provider–patient communication, and unintentional non-adherence, including forgetfulness, pathophysiological barriers, socioeconomic barriers (including lifestyle and patient factors), or healthcare resource limitations. Multifaceted, patient-tailored interventions that could improve adherence are discussed, including promoting health education, enhancing healthcare provider–patient engagement, and exploring alternative medical solutions and emerging technological advances. No single approach fits all; this review aims to deepen the understanding of intentional and unintentional non-adherence and to inform targeted interventions to empower patients, foster trust, and improve adherence for those with chronic conditions.

## 1. Introduction

With rises in life expectancies, the number of patients living with chronic diseases has increased [1]. As technology development expands, so do the range of treatment options, modalities, and routes of administration available to support these patients living with chronic diseases. Despite significant efforts in diagnostics, treatment decisions, and monitoring, the importance of treatment adherence is often underestimated or overlooked. As a result, it may not keep pace with the need to optimize chronic disease management and reduce the overall healthcare burden.

Non-adherence to prescribed treatment regimens in chronic diseases has serious consequences for the patient, contributing to morbidity and increasing mortality [2]. In chronic conditions, including hypertension, diabetes, and kidney diseases, poor adherence can lead to increased rates of organ damage and cardiovascular events [3]. In mental health conditions, poor adherence can worsen symptoms, reduce quality of life, and increase hospitalization and suicide rates [4]. Improving adherence is therefore essential for improving outcomes for patients and reducing preventable healthcare resource utilization [5,6], with the associated medical expenditure of non-adherence estimated to cost EUR 125 billion in Europe and USD 100 billion in the United States (US) each year [7].

In 2002, the World Health Organization (WHO) estimated that in developed countries, 50% of patients with chronic illnesses were non-adherent [8]. Twenty years later, these rates have not improved much; recent estimates for adherence in patients with multiple chronic conditions range from 44% (when electronically reported) to 77% (when self-reported) [9]. Medication adherence has been defined in several different ways, but can generally be considered as “the degree to which a patient follows healthcare provider (HCP) advice regarding timing, dosage, and frequency of medication use” [10,11] and is further defined by the WHO as “the extent to which a person’s behavior—taking medication, following a diet and/or executing lifestyle changes—corresponds with agreed recommendations from a HCP” [8]. It is well documented that adherence rates drop rapidly within the first year of treatment for many chronic conditions [12] and low adherence is one of the reasons suggested when findings from randomized clinical trials do not align with outcomes observed in the real world [13].

Non-adherence to treatment is a multifaceted and complex issue, influenced by intentional and unintentional factors. These factors may relate to the medical condition itself, medication characteristics, patient-specific circumstances, healthcare system limitations, socioeconomic barriers, or geographical challenges [8,14,15]. For instance, adherence rates to oral antidiabetic medications in type 2 diabetes vary significantly, ranging from 42% in Switzerland to 67% in Canada [16,17]. Similarly, adherence across other conditions also shows considerable variability: antihypertensive medications have adherence rates between 53% and 71% [18], antidepressants up to 50% [19], and medications for patients undergoing chronic hemodialysis between 3% and 80% [20]. The reasons for such variation may be related to how adherence is measured across the studies, with indirect methods such as self-reporting, pill counting, and electronic monitoring being open to bias [21]. Geographical variations in adherence may be attributed to differences in culture, healthcare systems, and medication affordability/access [22]. Adherence rates can also vary across different populations, with some being more at risk of non-adherent behavior. Adolescents are one population with particularly low adherences rates [8], with estimates of adherence ranging from as low as 30% to 70% for inhaled asthma medications [23], as low as 30% for pulmonary medications in general [8], and 52% to 70% for medications following liver transplant [24].

In this targeted, non-systematic review, we describe existing gaps in understanding treatment adherence and highlight its broad impact on patients, healthcare systems, and society. We aim to identify key issues impacting patients’ ability (whether intentional or unintentional) to adhere to prescribed treatments as well as risk factors to support assessing, monitoring, and discussing adherence with patients to facilitate shared care decision making. The purpose of this review is to deepen the understanding of the long-standing issue of patient non-adherence, as well as to highlight developing technologies, and emerging treatment options to inform targeted interventions and solutions.

## 2. Intentional Non-Adherence

**Figure 1:**
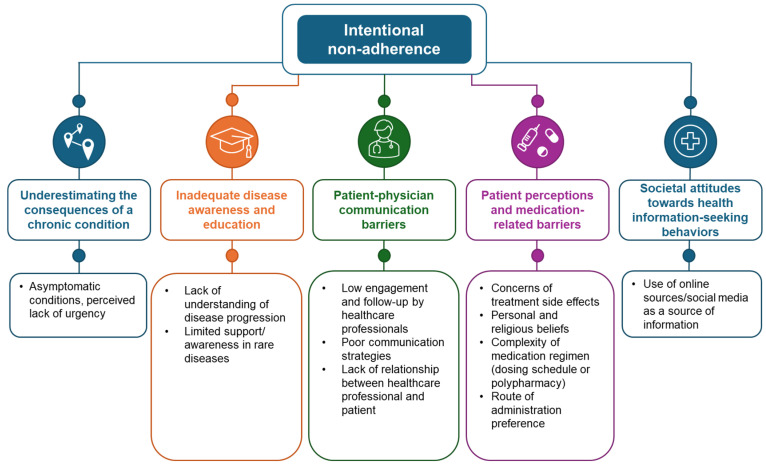
Types of intentional non-adherence.

Intentional non-adherence is where a patient deliberately alters or omits their treatment against recommendations. For the purpose of this review, we focus on medication adherence and reasons why patients intentionally deviate from their prescribed dosing regimen, with potential contributory factors described below and summarized in Figure 1. It is also important to acknowledge that adherence also relates to non-prescribed factors which are essential in disease control. Adherence to all forms of treatment, from lifestyle recommendations (e.g., in cardiovascular disease), dietary restrictions/fluid intake advice (e.g., in kidney disease) and device use (e.g., urinary catheterization for bladder control) are all at threat from intentional non-adherence.

### 2.1. Underestimating Consequences of a Chronic Condition

Patients may believe their condition is under control, either due to effective treatment or a lack of symptoms [25]. In chronic conditions, the latter is often associated with treatment initiation following an early or incidental diagnosis. Hypertension, for example, is frequently asymptomatic until complications occur, with reports of adherence to antihypertensive medications as low as 50% [26]. In children with urinary tract abnormalities, adherence to prophylactic antibiotics is also reported to be low (40%), despite the risks associated with urinary tract infections [27]. Similarly, in osteoporosis, where patients may not feel the effects of the disease until a fracture occurs, estimated adherence to oral bisphosphonates is as low as 19% at 2 years following treatment initiation [28]. Conversely, patients with severe symptoms pre-treatment, with a need for interventions or hospitalizations, tend to have higher adherence rates [29], as such experiences serve as drivers for adherence to medications [2,30].

### 2.2. Inadequate Disease Awareness and Education

A lack of patient awareness of the consequences of disease progression or complications of untreated chronic conditions can be an important contributing factor to poor adherence. Newly diagnosed and often asymptomatic hypertension, when left untreated, can lead to coronary artery disease, heart failure, stroke, chronic kidney disease, and vascular dementia [31,32]. Yet, up to 20% of patients do not fill their prescription, assuming the medication is unnecessary [25]. Whilst patient education is vital, healthcare resources often fall short in meeting the demands [2,30]. This challenge is even greater for patients living with rare conditions where limited disease awareness and restricted access to medications create additional adherence challenges [14].

### 2.3. Patient–Physician Communication Barriers

Low engagement from HCPs can contribute to patients feeling underinformed and lead to inconsistent follow-up [10]. In a survey of patients recently discharged from hospital, 21% reported that it was difficult to understand why they had been prescribed medications and over two thirds (68%) suggested a follow-up call would have improved adherence [33]. Studies in elderly patients suggest that patients who felt like they had poorer follow-up continuity with their HCP had greater uncertainty surrounding their medications, leading to poorer adherence [34].

Perceived poor communication between physicians and patients can also play a critical role in non-adherence. A meta-analysis of approximately 100 studies found that non-adherence was almost 20% higher amongst patients treated by physicians with poor communication ratings versus those who communicated well [35]. In one study of patients with chronic kidney disease, over half the surveyed participants claimed to have limited disease awareness, even though the majority had been given some form of counseling by their providers regarding their disease [36].

### 2.4. Patient Perceptions and Medication-Related Barriers

Intentional non-adherence could also be due to concerns related to the medication, such as fears of dependency or fears of side effects [37]. “Self-medication” can occur, where patients adjust the dosage or frequency without consulting a healthcare provider, often based on perceived improvement or worsening symptoms [38,39]. Personal beliefs can also play a role, such as religious or dietary views related to medicine excipients, as well as the potential impact on patients’ schedules or other lifestyle factors imposed by the treatment regimen [37,38].

Chronic medications can cause a range of unpleasant side effects ranging from gastrointestinal issues such as nausea, diarrhea or constipation, neurological issues such as headache and changes in mood, or physical discomfort in the form of weight gain, cough, dry mouth, and skin irritation. Fear of experiencing side effects is a common reason for non-adherence across multiple medications and conditions [30,38,39]. In diabetes, for example, interview-based studies frequently identify patients’ fears of side effects (either real or perceived) as a major reason for them not following their recommended treatment regimens [40,41,42]. In these cases, a patient’s concern for potential side effects can be more problematic than their concerns about the disease itself, with patients attributing unrelated physical ailments to their chronic medications [41]. In the case of diabetes medication, one study highlighted that for most treatments (83%) the side effects reported following use subsided within one month [40], highlighting the importance of clear communication about the likely duration of side effects to ensure long-term adherence.

Complex treatment regimens that involve taking many different medications per day [43] and/or multiple daily doses [44] or those that require specific timings or food restrictions [45] can be particularly challenging for adherence in certain patient populations. Furthermore, systematic reviews have identified that for medications which need multiple daily doses, adherence decreases as the number of daily doses increases [46,47]. In type 2 diabetes, one study evaluating adherence to oral hypoglycemic medications three years after initiation showed that patients’ adherence significantly decreased as the number of different medications increased [48]. Multiple drug–drug interactions are another difficulty in managing polypharmacy regimens safely, further complicating medication management and adherence rates particularly in elderly patients and those with complex conditions [49,50]. Some medications, such as oral anticancer medications, levothyroxine for hypothyroidism and metformin in diabetes, must often be carefully timed with food intake, due to issues with food–drug interactions, absorption or side effects [29,51,52] and patients often face difficulties keeping track of these complex schedules [29,45]. Treatment complexity is also a particular concern in rare diseases, where treatments often require orphan drugs and/or complicated regimens. For example, in distal renal tubular acidosis, commonly used treatments include sodium bicarbonate and potassium citrate, often prescribed to be taken between three times to up to five times daily [53]. This need for the constant administration of medication throughout the day will impact adherence, with one survey reporting that the majority of treating physicians believing their patients had suboptimal adherence to their regimens [53].

Route of administration is another factor impacting the successful initiation of treatment and subsequently continuing and reliably adhering to the prescribed regimen [8]. Whether oral or parenteral, the benefit of one route of administration over another will be dependent on individual patient factors and can be associated with pathophysiological or psychological barriers [8], which will be discussed in a later section. Provided there are no conflicting factors limiting drug bioavailability, orally administered treatments can be a desirable and perceived convenient option for many, especially among pediatric and adolescent populations [54]. However, oral medications often require more frequent dosing than injectable formations [29], thus further challenging the initial commitment to treatment. For instance, the administration of subcutaneous denosumab every 6 months in osteoporosis resulted in 50% adherence at 24 months, compared to 19% for those taking oral bisphosphonates, including risedronate, alendronate, ibandronate, and etidronate [28], which are often prescribed once-weekly [55]. Thus, close monitoring and counseling are required to avoid complications of poor disease control and to allow additional support or alterations in treatment where needed.

Finally, religious and non-religious dietary preferences can impact a patient’s willingness to take certain medications containing animal products and it is important that HCPs are conscious of potential conflicts and that alternatives are offered where available. Some religions might prohibit the use of bovine or porcine-derived products unless there is no alternative and where religious consensus is not clear, patients might be conflicted [56]. Examples of medications that include animal derivatives include heparinoids, hormones, and immunoglobins [56]; however, the shells of many capsule medications also contain gelatin, and lactose is a common component of tablets. Where available, starch-based capsules could be provided [57].

### 2.5. Societal Attitudes Towards Health Information-Seeking Behaviors

Limitations in healthcare systems because of factors such as time and resource pressures, as well as the popularization of patient-centered medicine has contributed to a shift from patients relying on a traditional trusting doctor–patient relationship to patients seeking alternative avenues for advice [58]. The growing influence of social media and online health forums can severely affect adherence, with one retrospective study of over 16,000 patients suggesting a negative relationship between reliance on digital content and medication adherence; patients who were solely reliant on digital sources had lower adherence and more negative perceptions of medications than those who relied on HCPs directly [59]. While legitimate patient organizations offer valuable support, almost a third of patients in a survey of 24,000 people with chronic diseases reported engaging in at least one behavior contradicting medical advice in the previous six months [38], such as skipping doses, altering doses or stopping taking their medications altogether.

## 3. Unintentional Non-Adherence

**Figure 2:**
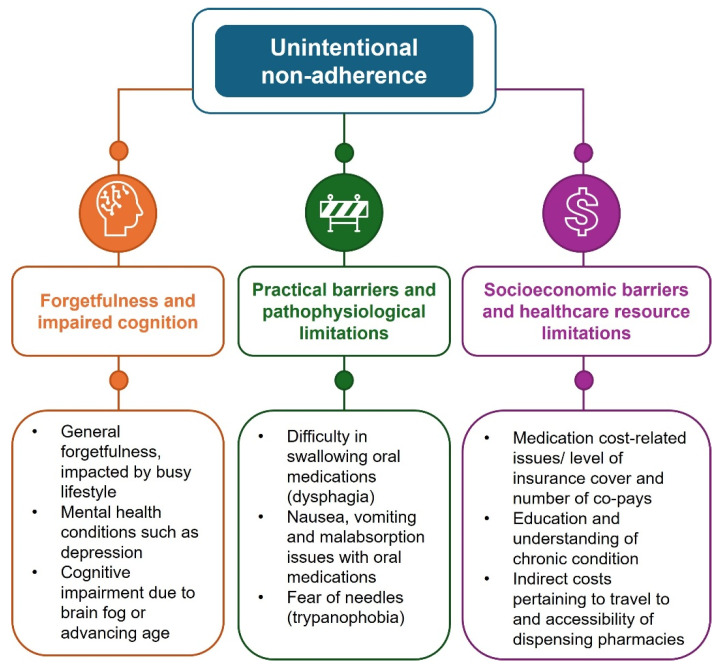
Types of unintentional non-adherence.

Unintentional non-adherence occurs when patients fail to follow prescribed treatments despite the willingness to do so. This can arise from a variety of causes, ranging from external distractions (such as a busy lifestyle, education, career), to patient factors (age, cognitive impairment, depression) or logistical barriers (refilling prescription on time, cost, geographical location, and access to specialized care; summarized in Figure 2).

### 3.1. Forgetfulness and Impaired Cognition

Forgetting to take medication is a major reason for non-adherence. In a large cross-sectional survey of approximately 24,000 adult patients in the US with chronic diseases, such as asthma, hypertension, diabetes, hyperlipidemia, osteoporosis, or depression, up to 62% of patients self-reported as having forgotten to take their medication [38]. Additionally, running out of medications or being careless when taking them was reported in 37% and 23% of patients, respectively, as the main reason for not adhering to their oral treatment regimens in the same previously mentioned study [38].

Mental health conditions such as depression or cognitive decline will also negatively impact a patient’s ability to adhere to their recommended treatments. In primary depression, approximately 50% of patients discontinue their antidepressant therapy early (within 6 months) [19]. When depression occurs alongside other chronic diseases, such as hyperlipidemia, hypertension, diabetes, and coronary heart disease, the likelihood of depressed patients being non-adherent is doubled compared to non-depressed patients [60]. Cognitive impairment, such as brain fog, represents another common cause of poor adherence and is a common manifestation of chronic diseases such as hypothyroidism [61] and cardiac disease [62] as well as rare chronic diseases like paroxysmal nocturnal hemoglobinuria [63]. A study of over 400 patients with hypothyroidism significantly associated treatment non-adherence with self-reported brain fog and highlighted the importance of interventions such as reminder tools to prevent forgetfulness and improve adherence [61]. In patients with heart failure, impaired cognitive function, specifically reduced memory, was a significant contributor to poorer treatment adherence [62]. Impaired cognitive function or decline is also a concern in managing treatment regimens for the elderly. Interview-based studies have highlighted that forgetfulness, or poor memory can be a cause of non-adherence in elderly patients [39,49] and that those who performed better on memory tests also had better adherence [49].

### 3.2. Practical Barriers and Pathophysiological Limitations

Unintentional non-adherence may also be a result of practical difficulties. Patients may intend to take their medications but be physically unable to. In the case of oral medications this could be due to difficulties in swallowing the medications themselves, arising from gastrointestinal disorders or other conditions that affect the ability to swallow or retain medication, or acute causes such as pregnancy or chemotherapy-related nausea [64,65,66]. Dysphagia is a symptom of many conditions, particularly neurological or nervous system disorders [67,68], and is reported to affect up to 40% of adults at some point in their lives [69]. Even in patients who do not have diagnosed difficulties in swallowing, fear of swallowing tablets or of a bad taste is a common barrier to adherence [70]. A survey of almost 700 patients, mostly aged between 60 and 89 years, identified 60% of respondents experiencing difficulties swallowing tablets/capsules and 69% failing to take their medication due to these difficulties [71].

Furthermore, patients who experience severe nausea, vomiting, or malabsorption may also struggle to maintain consistent dosing when taking oral medications, leading to suboptimal drug exposure and potential treatment failure [29]. Physiological or anatomical abnormalities can potentially compromise the effective absorption of oral medications. This could be through acute gastroenteritis, chronic conditions such as celiac disease, morphological and functional changes in the gastrointestinal tract related to advancing age, or variations in gastric emptying in pediatric populations [65].

The fear of needles, trypanophobia, can also be a significant barrier to adherence for patients requiring injectable medications, particularly those with chronic conditions necessitating frequent injections [72]. This fear can lead to skipped doses, anxiety, and even a complete avoidance of treatment, compromising disease management [72]. It affects around 20–50% adolescents and 20–30% of young adults to some degree [73]. In contrast to the physical barrier of dysphagia/difficulty swallowing, trypanophobia constitutes a mainly psychological barrier and could be mitigated by strategies related to the devices, as discussed in a later section.

### 3.3. Socioeconomic Barriers and Healthcare Resource Limitations

Socioeconomic factors, including financial constraints, limited access to healthcare, and lower levels of education, are another major driver in patients’ ability to adhere to medications [10]. One study in cardiovascular disease showed poorer adherence in neighborhoods of low socioeconomic status versus higher socioeconomic status [74]. In diabetes, higher income (>USD 60 k) was a significant predictor of good adherence to diabetes medications [75]. Food insecurity and housing instability were found in one meta-analysis to correlate with poor adherence, suggesting that patients prioritize these concerns over their medical conditions [76]. To manage costs, patients with insufficient coverage may delay treatment, reduce dosage, or avoid filling prescriptions altogether [38,39,77]. In a large US-based study, cost-related non-adherence was reported in 13% to 16% of patients with diabetes, cardiovascular disease or hypertension, with approximately 88% of patients citing affordability as the most common reason for this non-adherence [77]. Other socioeconomic factors, such as the level of education, can also impact adherence. Patients with higher education levels (above high school or graduate level) tend to have better adherence than those with lower education levels, in studies in both adults [75] and younger populations [78].

For countries without a government-sponsored healthcare system, such as the US, access to healthcare is largely dependent on the individual’s level of insurance coverage, whether private or publicly funded, which is greatly impacted by employment status and federal policy. According to 2022 US census data, 55% of the population had private employment-based insurance, 19% were covered by Medicaid (predominantly those over 65 years of age or with long-term disability), 19% by Medicare (state administered to low-income individuals), and nearly 10% by direct purchase [79]. The prohibitive costs of many medications may therefore further reduce adherence in uninsured populations, and out-of-pocket costs are significantly associated with poor adherence. A meta-analysis covering nearly 200,000 patients with chronic conditions in the US indicated an 11% increase in the chance of non-adherence in patients requiring to co-pay [80]. The price of insulin tripling in the US between 2002 and 2013 placed an unsustainable financial burden on patients, further reducing adherence rates [81]. Another study in diabetes demonstrated that adherence decreased by 11% with each additional USD 15 out-of-pocket cost per month [75]. In rare diseases, adherence challenges are even greater due to high medication costs and inconsistent insurance coverage [14,82]. The extent of financial barriers varies across conditions and healthcare systems, with local policies and funding structures significantly influencing medication access.

Poor medication adherence is also an issue in other countries without fully developed, government-sponsored health systems, including Pakistan and Nigeria. In Pakistan the healthcare system is reliant on out-of-pocket expenses to cover payment gaps, which will contribute to poor medication adherence [83,84]. One study of adherence to antihypertensive medication in the Islamabad region found that 73% of patients have uncontrolled hypertension. Half stated they were unable to afford their monthly medication and unaffordability was significantly associated with poor adherence [85]. In Nigeria, the National Health Insurance Scheme is accessible to less than 10% of the country’s population [86,87]. In a study of adherence in diabetic patients, over 60% were uninsured with the majority of these self-financed [86]. Adherence was significantly lower in uninsured patients versus insured patients, with medication costs thought to be a contributing factor [86]. In general, analyses of global trends in adherence to medications for chronic diseases such as diabetes and cardiovascular disease indicated that adherence tended to be poorer in low-to-middle income and non-Western countries and that socioeconomic factors such as lack of insurance and poor access to healthcare are linked to low adherence across multiple countries and continents [22,88].

Affordability of treatment also extends to the indirect costs of a treatment regimen, including costs of transportation to healthcare centers. In a study examining adherence in inner-city patients following hospital discharge, only 40% had filled their prescription on the day of discharge, 20% took up to 2 days and 18% up to 9 days; 65% of patients cited help with transportation to the pharmacy as a way of improving adherence [33]. Similarly, long pharmacy waiting times discourage adherence, and diabetes patients relying on retail pharmacies were twice as likely to be non-adherent compared to those using mail-order options [33,75].

## 4. Special Focus on Adolescent Populations

As mentioned above, adolescents are one population with particularly low adherence rates, with estimates as low as 30% for certain medications [23]. As for the general population, reasons for poor adherence in adolescents are multifactorial, arising from intentional and non-intentional causes.

### 4.1. Intentional Non-Adherence

As adolescent populations transition into adulthood and move from dependence on caregiver-managed regimens to self-management and making decisions about their own care, they may encounter issues with adherence related to underestimating their disease and inadequate disease awareness [24]. A poor understanding of their disease and the consequences of mismanagement may also make them more likely to engage in non-adherent behaviors [89]. Adolescents often report communication barriers with their HCPs, including perceptions of being treated like children and not being provided with an adequate understanding of their disease and its management [89]. Without open communication with physicians, patients may be reluctant to ask for further information on their condition [37,90] or may be hesitant to report adherence struggles and seek help with finding solutions, leading to undetected non-adherence. This can further result in serious and sometimes irreversible complications for many patients.

Another major barrier to adherence for adolescent populations is a perception of feeling different to their peers [24]. One systematic review of self-reported barriers to adherence in adolescents highlighted the importance of peer relationships and a desire to “appear normal” in adolescents remaining adherent [89]. Many patients reported concerns about feeling stigma from their peers regarding their disease as well as non-adherent behaviors such as not taking medications in order to “forget about their conditions” or “feel normal” [89]. There is also the impact of medication side effects to consider. For patients with nephropathic cystinosis, common side effects of cysteamine therapy include gastrointestinal issues as well as halitosis and body odor, which are noted to negatively impact patients’ ability to adhere and their quality of life [91,92]. Similarly, poor relationships between adolescents and their caregivers are frequent reasons for non-adherence [89]. This can be either through caregivers not delegating responsibility for their care to the adolescent, thus interfering with their transition to self-autonomy, or through adolescents feeling insufficiently supported by their caregivers to adhere to their regimens [89].

### 4.2. Unintentional Non-Adherence

Forgetfulness can be an issue for adolescents remaining adherent to their treatment regimens. A qualitative study designed to explore challenges in medication adherence for adolescents and young adults found remembering to take medications a major challenge for adherence [93]. Without the daily structure provided by a caregiver, adolescents and young adults are more prone to missing doses, especially with oral medications that require frequent, routine intake [24,89]. In contrast, injectable medications, which are usually administered less frequently and sometimes under clinical supervision, may help ease the transition by reducing the daily burden of self-management. In addition, younger patients may be more focused on education, career or life goals than their medical conditions [31,93] especially when they involve complicated regimens. Increasingly busy, modern lifestyles are likely to compound this issue.

## 5. Discussion and Strategies to Improve Adherence

**Figure 3:**
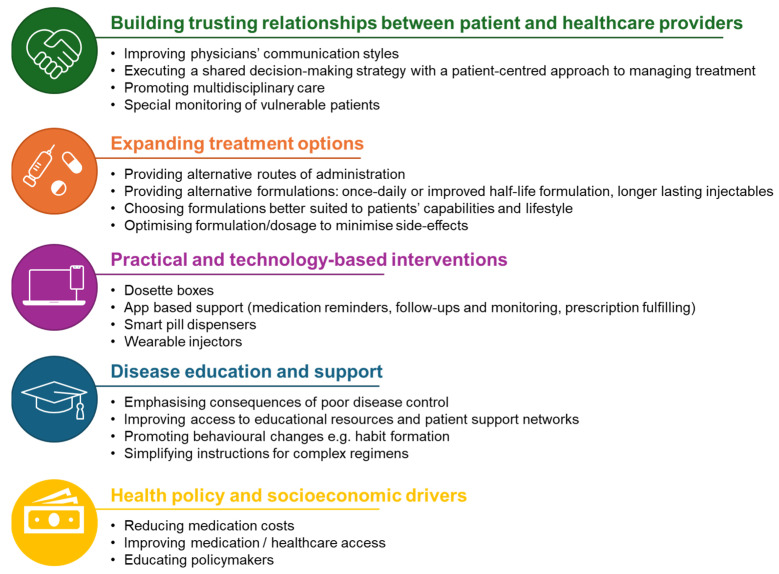
Strategies for improving adherence.

This review so far has shown the main contributors to low adherence, highlighting a need to implement strategies to identify and reduce the risk of non-adherence, maintain treatment schedules, and optimize disease control. With multiple factors and barriers contributing to non-adherence as discussed, optimizing treatment adherence requires a range of approaches (Figure 3).

### 5.1. Building Trusting Relationships Between Patient and Healthcare Providers

Any strategy chosen to improve adherence must involve HCPs. Strengthening HCP–patient communication is therefore key, as patients feeling able to discuss any concerns or difficulties regarding their medications with their HCPs will remove many major barriers to adherence [41,42,94]. HCPs need to provide clear and transparent information regarding the potential for side effects in order to gain informed patient consent; however, a balance must be made to prevent overwhelming patients with full disclosures of potential outcomes which could induce worry and non-adherence [95]. Meta-analyses have shown that patients are more likely to adhere to their treatments when their physicians have been trained in communication skills [35]. HCPs who are more engaged with their patients and communicate well are also more likely to identify factors which could impact a patient’s adherence and to recognize warning signs in non-adherent patients [2,3,30]. Special monitoring in vulnerable populations such as the young, elderly or those with mental health issues is key to ensure effective treatment and improve patient outcomes and extends to continuity of care upon discharge from a hospital setting [21,96,97]. Strategies to improve adherence should extend beyond the patient and their physician, involving pharmacists, nurses, and patients’ support networks, in a multidisciplinary care approach [2,98].

As the causes of non-adherence are multifaceted, one solution will not fit all patients. Promoting a shared decision making approach to choosing treatment regimens as previously mentioned will further build trust and communication between patient and HCP, contributing to patient understanding and improved adherence [90,94]. Shared decision making involves patients being actively involved in discussions about their treatment decisions, making it more likely to find treatment regimens that are personally manageable for them [94]. Personalized medication regimens, tailored to a patient’s specific needs, are much more likely to be adhered to. Such a strategy is in line with recommendations in guidelines from healthcare bodies such as the UK National Institute for Health and Care Excellence, suggesting the adaptation of a person-centered approach to medicines management that encourages adherence [99]. Effective implementation of shared decision making can be challenging in heavily burdened healthcare systems with limited resources, such as busy hospitals with a lack of private space for discussions, time-poor staff and limited training [100]. In such settings, shared decision making may compete with urgent clinical demands, reducing its practical implementation to a checkbox rather than a meaningful dialog. Changes in policies and management to address these issues, such as allowing quiet, dedicated spaces for HCP–patient conversations, allocated time for HCP–patient interaction, and emphasizing the importance of providing training would facilitate its adoption [100].

### 5.2. Expanding Treatment Options

Making a choice of a single treatment from a range of available options can create confusion and uncertainty but should be supported by shared discussions between patient and healthcare provider based on individualized considerations [58]. Route of administration may affect intentional or unintentional ability or willingness to reliably adhere to the medication regimen, potentially causing breaks in treatment that if not identified may lead to irreversible health consequences. This review has previously mentioned many factors that may impact the decision making process, including patient age, other comorbid conditions requiring treatment, lifestyle, socioeconomic factors and support networks, understanding of the disease and its progression, history of non-adherence, opportunities for regular follow-ups, strength of communication with HCPs, as well as patient involvement in their own disease management. Considering that medication non-adherence can often result from the physical characteristics of medicines, manufacturers should, whenever feasible, provide multiple formulations—such as liquids or solids—to better align with patient preferences [10,101]. Additionally, manufacturers can optimize medication strength, bioavailability, and dosing schedules to facilitate less frequent administration [10]. Kidney transplant recipients demonstrated preference (in 78% patients) for a once-daily prolonged-release tacrolimus formulation over a twice-daily immediate-release formulation, with high levels of patient adherence over 12 months [102]. With this less frequently administered prolonged-release treatment, 81% of patients reported it was easier to remember than the twice-daily dose; 68% cited a preference for the once daily formulation due to not having to remember the evening dose and 65% due to reduced pill burden [102]. Furthermore, the recent development of single-tablet combination therapies, combining multiple drugs into one formulation, has improved adherence and persistence in real-world studies [103]. In a large, real-world, claims-based study of antihypertensive medication in Korea, adherence was higher in patients receiving single-tablet combination therapy versus multiple-tablet combination regimens [104]. This effect was heightened with increasing age and number of pills, highlighting the benefit of simplifying treatment regimens in elderly patients [104]. Offering medications in both tablet and capsule forms and allowing patients to select their preferred formulation can further enhance adherence and patient satisfaction. This could extend to offering patients an alternative route of administration if problems arise with the initial route. For example, providing oral versus inhaled corticosteroids for chronic asthma could help adherence in developing nations due to lower costs [105], or providing options of topical versus oral non-steroidal anti-inflammatory medications for chronic joint pain, based on patient preferences [106].

We have discussed the issues of unintentional non-adherence related to pathophysiological and anatomical barriers impacting bioavailability related to the drug profile. As subcutaneous or intravenous therapies bypass the gastrointestinal tract, they often ensure more reliable drug delivery and absorption compared to oral options for patients prone to nausea or gastrointestinal intolerance, or those facing barriers such as dysphagia [29]. However, they come with their own barriers to adherence, including fear of needles and, where self-administration methods are not in place, the need to schedule regular clinic visits [29,107]. While no studies directly assess how needle size impacts needle fear, smaller needles and other innovations have been shown to reduce pain, a key factor in needle fear [108]. In patients with type 2 diabetes, 6 mm needles in insulin pens were associated with lower pain, greater patient satisfaction, higher patient adherence and overall better glycemic control compared to 8 mm needles [109]. In recent years, wearable injectors—devices attached to the body that automatically deliver medications—have also been presented as a robust solution to the challenge of delivering subcutaneous formulations in non-clinical settings [110,111]. This allows more patients the convenience of injecting at home and the design often allows less frequent injections, thus improving the patient’s experience, and potentially increasing adherence to therapies. Some wearable devices are designed with extremely small, non-visible needles, which remain hidden from the patient throughout their use, helping to mitigate any patients’ concerns surrounding needle fear [108,111]. Among non-device-related strategies, distractions during the injection procedure, relaxation techniques, and education on medical equipment were also perceived as helpful [73,108]. The availability of a multi-disciplinary team is vital to optimize confidence in drug administration where technical training is required, for example, in the self-administration of subcutaneous treatment regimens. Additional patient support programs exist to further support patients in practical considerations, and in combination with advice from HCPs, have been shown to improve confidence in perceived complex medication delivery [112,113]. Awareness and signposting of such programs is therefore a key strategy in improving adherence.

Finally, medications should also be carefully chosen to minimize the potential burden of side effects for patients [2,10]. This could be combined with better follow-up and monitoring procedures, ensuring that medications are frequently reviewed and that there is open communication about the possible severity and duration of side effects [2], including reassurances that current side effects may not continue indefinitely [40,114]. Objective measures of disease control or improvement are also likely to motivate patients to maintain their treatment regimen [37].

### 5.3. Practical and Technology-Based Interventions

For patients with complex medication needs, such as those requiring frequent dosing or managing polypharmacy for comorbid conditions, the simple use of a multicompartment pill organizer or dosette box can be beneficial, with the caveat that assistance with weekly organization and careful monitoring is required in vulnerable or elderly populations [115]. More recent developments in technology-based interventions could further personalize patients’ treatment regimens, making them easier to manage [116]. This could involve services which actively remind patients when to take their medicines or refill their prescriptions, for example, targeted medication reminders sent through devices such as mobile phones [37,117] or specific disease-related applications that manage their daily medication regimen [118]. The use of electronic monitoring devices, such as electronic pill dispensers or smart pill bottles could help HCPs track the patterns and frequency of medication use [3]. For patients with asthma, for example, use of metered dose inhalers can effectively track medication use, with one study reporting increases in adherence from 29% to 54% after a five-week intervention [119]. Finally, patients could benefit from telemedicine follow-ups or remote monitoring [2,10,116]. A systematic review of telehealth interventions in patients with affective disorders (including telephone calls, video consultations, and internet-based self-management programs) found that the majority (59%) of interventions had a positive effect and improved adherence [120]. However, increasing reliance on technology to monitor or improve adherence may disadvantage those with poor digital literacy or access to smart phones and other technologies. Certain populations, such as the elderly or those from lower socioeconomic backgrounds, may be left at a higher risk for non-adherence.

### 5.4. Disease Education and Support

Promoting better disease education in patients, including the need for treatment, potential complications and outcomes of poor disease control is a key approach to encourage adherence [2,75]. This could be either providing more resources for increasing disease awareness such as directing patients to trustworthy sources of information online, or providing simple, clear instructions for medication regimens, especially in the case of children, young adults, and the elderly [2,30,96]. Behavioral changes could also be promoted, such as highlighting the benefits of habit formation to help memory or strategies such as motivational interviewing [37]. Patients could be encouraged to associate taking their medications with specific environmental cues. In a study of patients with epilepsy, those who linked taking their medication with a specific time, place or activity (such as brushing teeth) were more adherent to antiepileptic medication than those who did not (93% of doses taken vs. 55%) [37]. Interventions could also be designed to address and improve patients’ negative beliefs regarding their medications. One such program in stroke survivors which included a component designed to modify patients’ beliefs regarding medications, resulted in a 10% increase in adherence for those completing the program versus those who did not [121]. Similarly, participants in an online intervention providing support to overcome perceptual barriers to taking medication in inflammatory bowel disease showed significantly fewer doubts about their treatment and higher adherence than those who did not take part [122]. In addition, the medications themselves could be optimized to improve adherence. This could include promoting simplified regimens, such as once-daily formulations [3] or medications with a longer half-life, which are more forgiving for patients who unintentionally miss or delay a dose [30].

### 5.5. Health Policy and Socioeconomic Drivers

Reducing medication costs would help to improve adherence in patients limited by socioeconomic factors. The use of generic medications, which are generally more affordable, should be encouraged [123]. More cost-effective formulations could also be explored, such as the use of single-tablet combination therapies, which can be cheaper than the use of multiple single-component products [103]. One analysis of adherence in cardiovascular disease showed that policy interventions to reduce patients’ out-of-pocket costs or to improve their insurance coverage were significantly associated with improved adherence [124]. Interventions to improve patients’ access to medications could also be beneficial, for example, by extending clinic/pharmacy opening times and increasing the ease of obtaining prescriptions [30,33] or by providing transportation assistance to appointments for those with disabilities [33]. Such interventions could be underpinned by initiatives aimed towards policy makers to improve their awareness of the impacts of high costs and poor medication availability on health outcomes [10]. The impact of geographical inequalities in access to healthcare should be addressed as rates of adherence tend to be poorer in low and middle-income countries, especially those without access to universal health coverage [22,88].

## 6. Conclusions

Many patients underestimate the consequences of poor adherence, especially in chronic conditions where symptoms are mild or seem to be controlled by current treatments. Non-adherence leads to poor disease control, suboptimal outcomes, and serious health risks. Causes vary widely, making it difficult to predict or address at treatment initiation. Effective management requires open patient–HCP discussions to identify adherence barriers, personalized treatment, and addressing unintentional disruptions. As patients juggle busy lives and seek control over their health, strategies like remote consultations, medication simplification, reminders, and self-administration help maintain adherence. Emerging technologies—artificial intelligence, predictive analytics, and digital health tools—offer opportunities for further personalization. Supporting patients with verified education, patient programs, and strong HCP relationships is essential. No single approach fits all, but tailored, scalable interventions and innovative, patient-centered strategies will empower individuals, foster trust, and drive better adherence and ultimately, outcomes.

## Data Availability

Data sharing is not applicable.
